# Utility of N-acetylcysteine in Non-Acetaminophen-Induced Liver Injury Secondary to Influenza A Infection

**DOI:** 10.7759/cureus.70900

**Published:** 2024-10-05

**Authors:** Esha Jain, Rishab Alex, Theresa Coutinho, Navjot Narula

**Affiliations:** 1 Family Medicine, Cooper University Hospital, Cooper Medical School of Rowan University, Camden, USA; 2 Family Medicine, Ross University School of Medicine, Bridgetown, BRB; 3 Medicine, Cooper Medical School of Rowan University, Camden, USA

**Keywords:** acute liver injury, influenza a hepatitis, influenza a infection, nac, n-acetylcysteine, non-acetaminophen liver injury

## Abstract

Influenza A, which belongs to the Orthomyxoviridae viral family, is a known causative agent of respiratory illness and systemic inflammation. Annual influenza immunizations are crucial in reducing the incidence and severity of the flu. Intravenous (IV) N-acetylcysteine (NAC) is a pharmaceutical agent indicated for hepatic injury, particularly to address oxidative stress and inflammation secondary to acetaminophen toxicity. The authors present a case of a young female with acute liver injury and impending liver failure in the setting of viral influenza A infection, successfully treated with IV-NAC.

## Introduction

Influenza (Flu) A virus is a non-hepatotropic single-stranded RNA virus transmitted via respiratory droplets and through direct contact with infected mucus membranes of others [1,2]. Influenza is a very common seasonal illness; Tokars et al. report the seasonal incidence of influenza in the USA in 2018 as approximately 5% to 20%. Annual immunization against the flu is the single most important way to prevent it, along with good hygiene precautions. The immunization is recommended for populations of six months of age and older [3]. Untreated influenza A can lead to viral progression ultimately invading multiple organs with extra-respiratory complications such as encephalopathy and liver disease [2]. Classically leads to a respiratory infection with rare occurrences of hepatic involvement, accounting for less than 3% of all cases [1]. While the pathophysiology behind hepatic insult is unclear, it has been shown that hepatocyte damage is mediated by influenza-specific T-cells interacting with Kupffer cells in the absence of the Flu A antigen in the liver [4].

Acute liver failure (ALF) is a critical multi-systemic condition characterized by the rapid deterioration of liver function, culminating in hepatic encephalopathy. ALF is stratified into hyper-acute, acute, and subacute stages based on the time frame between the onset of jaundice and the development of encephalopathy, with ALF defined as occurring within less than 26 weeks [5]. N-acetylcysteine (NAC) is a pharmacological agent predominantly studied and utilized in the management of acetaminophen-induced hepatotoxicity. The therapeutic rationale for NAC lies in its systemic mechanism of action, which includes the augmentation of glutathione levels, reduction of oxidative stress, and attenuation of pro-inflammatory cytokine production [6]. The clinical importance of NAC is increasingly recognized in literature, particularly in cases of severe influenza A infection that has escalated to systemic inflammation, compromised organ perfusion, and contributed to hepatic injury [7].

The authors present a case involving a generally healthy young female patient who developed life-threatening acute liver injury, secondary to an influenza A infection. This report demonstrates the potential utility of intravenous (IV) NAC in managing acute impending liver failure. Additionally, we emphasize the importance of maintaining up-to-date vaccinations to help with a decrease in infection rate and thereby its complications.

## Case presentation

This is a 49-year-old female with a relevant history of daily acetaminophen and alcohol use (two shots per night) for the past five years, one pack-year smoking history, anxiety disorder, and sea moss and black seed oil supplement use who presented to Cooper University Hospital with a five-day history of persistent nausea and vomiting in the setting of influenza A, diagnosed three days ago at her workplace. The patient was not vaccinated for influenza. 

She reported chills, productive cough, chest tightness, decreased appetite and oral intake, generalized diffuse abdominal tenderness, and intermittent loose, yellow-tinged stools for the past week.

Vital signs in the emergency department were as follows: blood pressure: 109/70, pulse: 75, temperature: 98.2°F (36.8°C), respiratory rate: 16 breaths/minute, height: 1.676 m, weight: 79.4 kg (175 lb), and SpO_2_: 99%. The physical exam revealed the patient to be in acute distress, with pale skin but no jaundice, diaphoretic, RUQ abdominal tenderness, no peripheral edema, and no focal deficits on the neurological exam.

Upon workup, she was found to have severe transaminitis with hepatocellular predominance with ferritin serum at 45,000, AST in the 13,000s, and ALT in the 10,000s, as seen in Table 1 and Table 2. Other remarkable labs include influenza A+, EBV carrier, and an ammonia level of 48. RUQ US, CT abdomen/pelvis, venous US of hepatic vasculature, serum alcohol, urine drug screen, and acetaminophen levels were negative. Viral, autoimmune, and ischemic causes of liver injury were negative as seen on serum hepatitis panel, CMV, VZV, EBV, HSV, herpes antigen, ceruloplasmin, alpha-1 antitrypsin, anti-smooth muscle antibody, anti-nuclear antibody, and anti-mitochondrial antibody. Liver panel labs were initially monitored every eight hours until a significant decrease in transaminitis was observed. She was deemed medically safe for discharge on day three of admission with close follow-up with her primary care and gastroenterology physicians along with repeat liver enzyme and ferritin levels. Follow-up labs from subsequent emergency room visits demonstrated the resolution of transaminitis. 

**Table 1:** Liver function tests from admission to discharge

Component	Normal lab ranges	1/5/24, 11:29 am	1/5/24, 5:28 pm	1/5/24, 6:45 pm	1/6/24, 12:30 am	1/6/24, 7:49 am	1/6/24, 2:37 pm	1/7/24, 12:22 am
Alkaline Phosphatase	39-117 u/L	140	126	119	102	122	115	120
Bilirubin Total	N: 0.0-1.2 mg/dL	2.4	2.0	1.6	1.3	1.8	2.0	2.2
Bilirubin Direct	N: 0.0-0.3 mg/dL	1.2	1.0	0.7	0.5	0.9	1.3	1.4
ALT (SGPT)	N: 6-45 U/L	1,962	2,773	3,067	2,625	2,676	2,014	1,763
AST (SGOT)	N: 10-35 U/L	10,420	13,148	13,834	12,740	9,530	4,958	2,614
Albumin	N: 3.8-5.3	4.7	4.1	4.0	3.8	3.9	3.4	3.6
Protein	N: 6-8.5 g/dL	7.8	6.9	7.0	6.0	6.3	5.5	5.9

**Table 2:** Ferritin levels admission to discharge

Component	Normal lab ranges	1/5/24 (4:43 pm)	1/7/24 (2:07 am)
Ferritin	10-232 ng/mL	54,002	6,484

## Discussion

Influenza is an acute, self-limited respiratory illness with viral replication occurring exclusively in the respiratory epithelium. Common complications include myocarditis, pericarditis, renal failure, myositis, and meningoencephalitis. Influenza A binds to the respiratory epithelium by utilizing surface glycoconjugate sialic acid residues and invades through endocytosis, releasing viral RNA for replication and translation. Newly synthesized virions bud from the host cell, facilitated by neuraminidase, to infect adjacent cells [8]. Although uncommon, Flu A can invade hepatocytes, leading to hepatic inflammation and potential hepatic dysfunction or failure, characterized by elevated hepatic enzyme levels (AST greater than 40 U/L and ALT greater than 45 U/L), as seen in our patient. Hepatic involvement is observed in approximately 3% of all seasonal and pandemic influenza A outbreaks [1].

NAC, originally known as the primary treatment for liver failure due to acetaminophen toxicity, is increasingly used for other causes of acute liver injuries. Recent studies indicate NAC's beneficial role in non-acetaminophen-induced acute liver failure (NAI-ALF). NAC primarily scavenges free radicals, replenishes glutathione, and provides sulfate to prevent hepatic damage. Additionally, its anti-inflammatory, antioxidant, and vasodilating properties enhance hemodynamics and tissue perfusion [7]. Due to its systemic mechanisms, NAC has been utilized in various small trials of NAI-ALF, resulting in favorable outcomes. Various animal models support the therapeutic index of NAC in cases of NAI-ALF, demonstrating its ability to reduce key contributors to ALF, such as oxidative stress and inflammation. Furthermore, a study conducted by Mumtaz et al. supported the use of NAC, reporting a survival rate of 47% in NAI-ALF. NAC effectively reduced mortality in NAI-ALF and increased oxygen utilization, contributing to improved liver function in patients [9].

The pattern of elevated transaminases observed in this particular case mirrors that of viral and autoimmune hepatitis. Concurrently, a chronic history of acetaminophen and alcohol use potentially suggests drug- and alcohol-induced liver failure. However, the absence of autoimmune and viral markers highlights the impact of Flu A infection. An unremarkable abdominal US helps rule out fatty liver disease and alcoholic cirrhosis. Furthermore, prompt exclusion of hemophagocytic lymphohistiocytosis (HLH) is achieved by the absence of cytopenias, splenomegaly, hypertriglyceridemia, and elevated CD25 [10]. Given the prompt recovery of our patients, transaminitis and ferritinemia with IV-NAC, coupled with a normal hepatic US, we attribute the acute rise in this patient's liver enzymes primarily to influenza A infection. Although acetaminophen use, alcohol, and herbal supplements may have contributed, the patient's rapid improvement in liver function tests with IV-NAC strongly suggests that the acute liver injury was secondary to the influenza A infection.

While the use of NAC in NAI-ALF remains controversial, future research should focus on establishing clear management guidelines, including dosing and duration of NAC treatment for NAI-ALF. The stable safety profile of NAC underscores the need for further research to establish its use in a broader spectrum of liver injuries. The authors also want to emphasize the importance of routine health maintenance with the annual inclusion of influenza immunizations. Multiple evidence-based studies show that the flu vaccine greatly reduces the illness severity in individuals who are vaccinated but still contract the virus. The CDC reports that flu immunization not only prevents thousands of hospitalizations annually but also allows for those with underlying chronic conditions, such as COPD, asthma, or diabetes, not to experience worsening progression if infected [11]. Ultimately, vaccination against influenza is instrumental in reducing illnesses, hospitalizations, and deaths. 

## Conclusions

Commonly known for its role in managing acute liver injury secondary to acetaminophen toxicity, IV-NAC can display utility in non-acetaminophen hepatic toxicity as demonstrated by this patient's case. This case emphasizes the important role that vaccinations have as an additional barrier to protection from seasonal illnesses especially in high-risk work settings.
